# Improving Micro– and Macro–Algae Speciation Mapping for Management: A Field‐Validated Sentinel‐2 and Sentinel‐3 Intercomparison in the Indian River Lagoon

**DOI:** 10.1029/2026GH001817

**Published:** 2026-10-18

**Authors:** McKenzie L. Woodman, Edith A. Widder, Warren H. Falls, Joseph D. Ortiz

**Affiliations:** ^1^ Spectral Evolution Inc. Haverhill MA USA; ^2^ Earth Sciences Department Kent State University Kent OH USA; ^3^ Oceanic Research and Conservation Association Vero Beach FL USA

**Keywords:** satellite remote sensing, harmful algae blooms, cyanobacteria, seagrass, dinoflagellates, VPCA spectral decomposition

## Abstract

In recent years, the health of the Indian River Lagoon (IRL) on Florida's east coast has fluctuated due to recurring harmful algal blooms (HABs). These events typically involve cyanobacteria, dinoflagellates, or *A. lagunensis* (brown tide) causing water‐quality degradation and seagrass decline. The IRL is prone to HAB formation, and they have been observed more commonly in recent years. Although HABs can be non‐toxic, they can threaten critical primary producers like seagrass and destabilize sediments. Due to their impact, it is critical to improve methods of water quality and algal bloom monitoring. To do this, we apply a multi‐image varimax‐rotated principal component analysis (VPCA) to images from a harmonized Sentinel‐2 A/B multispectral imager (MSI) data set spanning three years (2020–2022). Field collected hyperspectral data and a coincident Sentinel‐3 A/B OLCI image is used to further validate the multi‐image VPCA. The VPCA method employs visible derivative water surface reflectance and unmixes the spectral signal to identify orthogonal components related to divisions of algae, suspended sediments, and seagrass. This advances water quality monitoring by improving unmixing and identification of optical components over time. This identification is supported by high correlation to a spectral library, field observation, published observations, and validation across sensors of varying resolutions. Through this research we find confident identification and mapping of in‐water components related to cyanobacterial (component 1 *r* ^2^ = 0.865) and dinoflagellate blooms (component 4 and 5 *r* ^2^ = 0.986 and *r* ^2^ = 0.978), and seagrass beds (component 3 *r* ^2^ = 0.916) with 10 m spatial resolution through time improving water quality monitoring and root cause identification.

## Plain Language Summary

The Indian River Lagoon (IRL) in Florida has experienced fluctuating water quality in recent years due to harmful algae blooms (HABs). These blooms are often caused by species of algae like cyanobacteria, brown tide, and dinoflagellates. An algae bloom can grow so dense that it can block sunlight from other species like seagrass and cause other harmful effects to the ecosystem. Parts of the IRL have high nutrients caused by sewage pollution that enable HABs to occur. Due to HABs becoming more common, a better technique for monitoring water quality and algae extent is necessary to assist environmental agencies and decision makers. To improve these monitoring systems, we use a multi‐image remote sensing method (VPCA) to process 10 m spatial resolution Sentinel‐2 A/B images over a 3‐year period. By doing this we can unmix spectrally complex reflectance data, identify micro‐ and macro‐algae, and develop time‐series maps of water constituents.

## Key Points

- A novel remote sensing method to characterize constituents in optically complex water such as cyanobacteria, dinoflagellates, and seagrass
- These methods can be translated into different areas and sensors to provide active mapping of water quality constituents
- Methods validated using field‐based hyperspectral data, coarser spatial resolution Sentinel‐3 images, and synchronous water sample data

## 1 Introduction

The Indian River Lagoon (IRL) is a 156‐mile‐long estuary along Florida's east coast, encompassing roughly 40% of the state's eastern shoreline. The contributing watershed for the IRL is 2,284 square miles (5,915.5 square km) with a surface area of 353 square miles (914.3 square km). The watersheds land uses are primarily urban land, agriculture, and range (Bricker et al., 2008; Lapointe et al., 2015). The lagoon is a brackish estuary with an average depth of 4ft (St. Johns Water Management District, 2007). The estuary contains 27% of Florida's salt marshes and an annually estimated $30 million USD fishing industry. The IRL is home to vast biodiversity, containing 2,100 species of plants and 2,200 species of animals (St. Johns Water Management District, 2007).

In recent decades, the IRL has experienced frequent harmful algal blooms (HABs) dominated by cyanobacteria, dinoflagellates, and the pelagophytes *Aureococcus anophagefferens* and *Aureoumbra lagunensis* (brown tide). These blooms have caused significant ecological and economic damage to coastal ecosystems worldwide (Koch et al., 2014). The major bloom event in 2012 within the Mosquito Lagoon (north of study area) and northern IRL marked the first recorded large‐scale brown tide in the region, primarily involving *A. lagunensis* (Phlips et al., 2015). Since then, brown tide, cyanobacteria, diatoms, and dinoflagellates have continued to persist in the IRL, posing ongoing environmental and economic threats.

The conditions of the IRL make it prone to HAB formation. The IRL is poorly flushed with estimated mean water half‐lives (i.e., 50% exchange) of 107 days in the Northern IRL, and 156 days in the Banana River Lagoon (Phlips et al., 2015) and has proven to be heavily contaminated by human wastewater and bloom‐forming macroalgae which increase during the wet season (Barile, 2018). Also, anthropogenic nitrogen input to coastal regions has been shown to drive phosphate limitation, enhancing phytoplankton access to phosphate bound in organic matter (Mahdy et al., 2022). The IRL gets an average of 50 inches (127 cm) of rain each year. Salinity and tidal mixing are highest at ocean inlets and higher rainfall causes a decrease of salinity in certain areas and lower amounts of rainfall will create more saline conditions (Phlips et al., 2020). This study focuses on a northern portion of the IRL containing the Banana River, and North IRL basins. The study area is shown in Figure 1 and primarily covers the Banana River. The Banana River has fewer freshwater inflows and thus higher salinity than the more southerly areas of the IRL (St. Johns Water Management District, 2007). The benthic cover in the lagoon bed is primarily sand and silt, with shelly hash found in areas with greater current velocity, and seagrass meadows. About 10% of the lagoon bed is covered in muck, a fine‐grained, organic‐rich clay with high water content that is associated with anoxia (Bradshaw et al., 2020). This muck typically accumulates in the deepest parts of the lagoon and near the mouths of tributaries, where freshwater discharges carry sediment and contaminants from agricultural and urban runoff (Bradshaw et al., 2020). Sediment resuspension events can further reduce water clarity and release nutrients back into the water column, promoting HAB formation.

**Figure 1:**
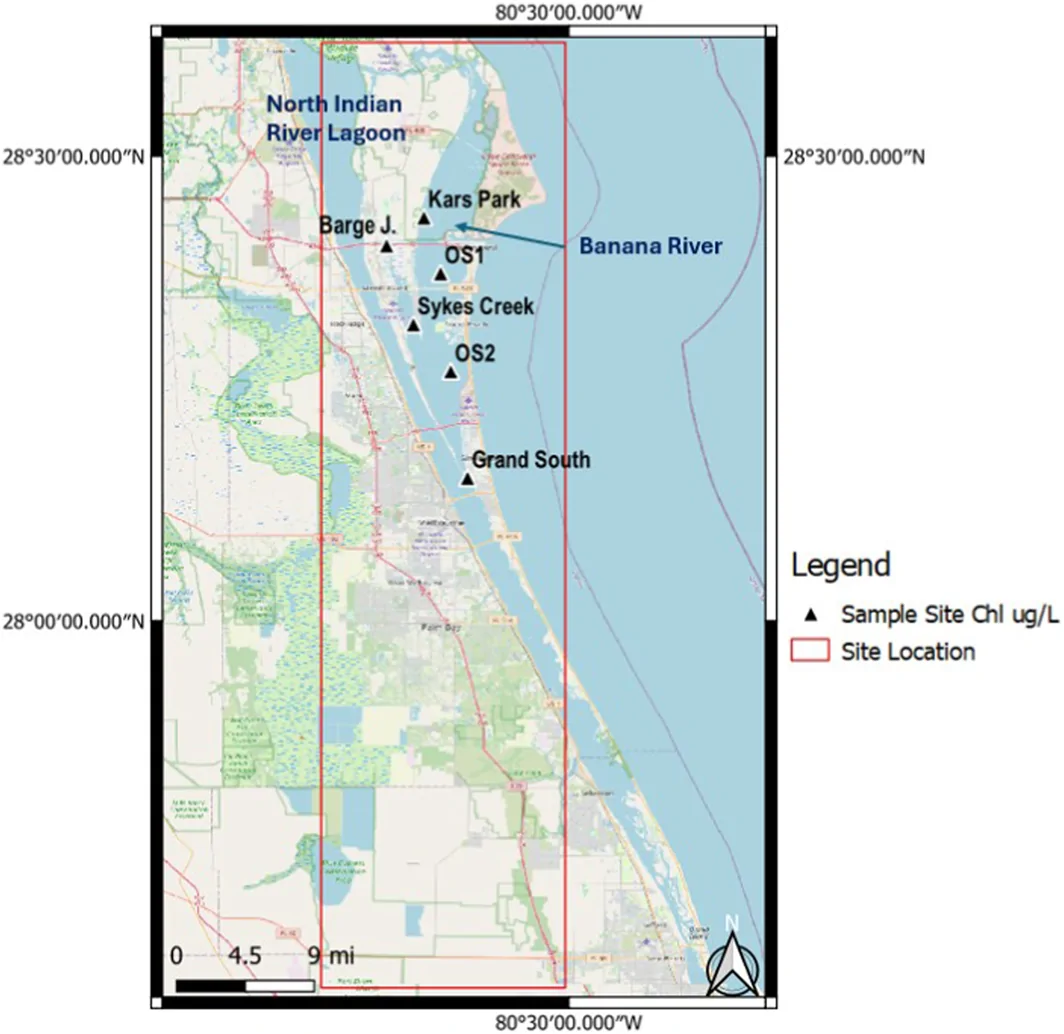
Area of interest map of the northern Indian River lagoon. Locations of in situ water samples for cell count and biovolume data as well as hyperspectral Analytical Spectral Devices™ measurements, Yellow Springs Instrument EXO2 sonde, and Secchi depths on August 1st and 3rd, 2022. Sites include Kars Park, Barge Junction, Offshore 1 “OS1”, Sykes Creek, Offshore 2 “OS2”, and Grand South. These are locations of piling‐mounted Kilroy instruments for water measurements apart from OS1 and OS2 which are open water stations.

In addition to anthropogenic activities that drive elevated nutrient loading, projected climate change is expected to further complicate coastal restoration and nutrient management efforts (Chen et al., 2026). Rising atmospheric CO_2_ concentrations and associated ocean acidification may also alter phytoplankton community structure and contribute to shifts in the relative dominance of different algal groups. For example, warmer and more acidic conditions are projected to favor some dinoflagellates, potentially increasing the frequency and intensity of harmful blooms (Gao et al., 2019). One of the most severe consequences of HABs in the IRL has been the collapse of seagrass populations, which has declined to an average percent cover of only ∼4% (Lapointe et al., 2015, 2020; Morris et al., 2022). The decline of the seagrass population threatens a critical food source for the endangered Florida manatee and there is a need to understand how HAB impacts on macroalgae effects foraging ecology (Long et al., 2024). The IRL has experienced a long‐term trend of seagrass decline and has contributed to worsening water clarity (Brewton and Lapointe, 2023, 2025). Between 2011 and 2017, the IRL experienced catastrophic seagrass losses following a series of unprecedented HABs, including persistent brown tides dominated by *Aureoumbra lagunensis* (Lapointe et al., 2020). From 2011 to 2019, approximately 58% of seagrass cover was lost, with canopy edges retreating shoreward into shallower waters, showing significant declines in percent cover (Morris et al., 2022). Seagrass meadows are essential to the lagoon's ecological integrity, providing food for manatees and sea turtles, nursery habitats for fish, and stabilization of bottom sediments. Economically, their value has been estimated at $4,600 per acre per year (Lapointe et al., 2015). Given their ecological and economic importance, the rapid decline of seagrass in the IRL underscores the urgent need for improved water quality monitoring and HAB detection systems.

Monitoring efforts in the area have used traditional methods with physical field studies and imaging from aircraft. Remote sensing efforts have utilized coarser spatial resolution satellites like Sentinel‐3 OLCI and have been limited in their time series analysis by compiling single day component analysis and correlating components to one another. These methods have limitations in cost, coarse spatial interpretability, and ability to apply these over large time series, we aim to improve the ability to interpret components over time and improve spatial resolution, using primarily open‐source tools.

We hypothesized that despite the lower spectral resolution of Sentinel‐2 MSI, application of a multi‐image varimax‐rotated principal component analysis (VPCA) will isolate spectral components whose spatial and temporal patterns correspond to dominant algal groups and benthic vegetation in the IRL, while providing substantially improved spatial resolution, and time series analysis for mapping these features.

To test this hypothesis, we applied the multi‐image VPCA spectral decomposition method to Sentinel‐2 MSI images from the start of 2020 to the end of 2022 and used observational field data and a Sentinel‐3 OLCI image to further validate the readings. The method numerically unmixes water spectral reflectance derivative signals and allows one to differentiate constituents in the water column. We have collected field validation data in the form of water samples analyzed for cell count, biovolume, Secchi depth, sonde data, and samples collected with a hyperspectral handheld spectroradiometer. The field spectral data was used in concert with field sensors of the Kilroy Continuous Water Monitoring System operated by the Oceanic Research and Conservation Association (ORCA), to validate the optical sensor readings. This enabled us to analyze the efficacy of the imaging, and to correlate different conditions with the occurrence of blooms. This research follows past research using the multi‐image VPCA method on Sentinel‐3 OLCI data with a significantly smaller data set shown in Judice et al. (2020). Judice et al. (2020) demonstrated effective identification of HAB's primarily identifying a large *Aureoumbra lagunensis* bloom during their study period. Applying VPCA in our study did not identify brown tide due to its lack of presence. Instead, during this study, the method identified other micro‐ and macro‐algae present including dinoflagellates, cyanobacteria, and seagrass. Sentinel‐2 A/B MSI was chosen for this study due to its increased spatial resolution at 10 m pixel size compared to Sentinel‐3 OLCI's 300 m resolution. The high spatial resolution of Sentinel‐2 provides an opportunity to understand spatial details of floating algae (Liu et al., 2021). Sentinel‐2 A/B MSI's coarser spectral resolution and temporal frequency is addressed by validating the results using Sentinel‐3 OLCI data, continuous hyperspectral Kilroy station data, and hyperspectral field data for comparison, as well as a 3‐year sample period yielding a significant sample size of 109 clear Sentinel‐2 A/B MSI images.

Through this study, we demonstrate the capability of the multi‐image VPCA method to unmix Sentinel‐2 A/B MSI reflectance signals and accurately identify and map HABs, and seagrass within the IRL. Components related to suspended sediments were also identified but further analysis is needed to fully differentiate sediment mineralogy given the lower band count of the MSI sensors. This research advances division‐level identification, spatial mapping of HABs and macroalgae, as well as accessibility using predominantly open‐sourced tools except for ENVI/IDL which was used for the Sentinel‐3 validation. By doing so, this study enhances HAB monitoring efforts and provides communities with improved data and analytical tools for integration into early warning systems and supports more effective responses for public health protection and mitigation of potential bloom drivers.

## 2 Materials and Methods

### 2.1 Varimax‐Rotated Principal Component Analysis

VPCA is an unsupervised soft‐classification technique that makes use of all coherent, linearly correlated spectral information present in the image (Ortiz et al., 2017, 2019). To conduct this study the VPCA method was applied to hyperspectral field data, Sentinel‐2 MSI, and Sentinel‐3A OLCI derivative spectral data sets. The VPCA decomposition method enables unmixing of constituents that are present in the water column, yielding component loadings and calculated component score maps that are associated with various types of algae, pigments and minerals. A VPCA is an eigenvalue‐eigenvector decomposition that partitions an image hypercube into independent signals generated by the underlying constituents that are present in the scene (Ortiz et al., 2013, 2017, 2019). It reduces the dimensionality of multivariate data sets by decomposing the integrated spectral signatures into orthogonal axes that each account for a portion of the total signal variability (Judice et al., 2020; Ortiz et al., 2019). Separate forward PCA rotations were conducted on each of the correlation matrices of the derivative‐transformed spectral data sets. Using the center‐weighted first derivative of the reflectance spectrum as the basis for the spectral decomposition, this removes background scattering and accentuates absorption features in the visible and NIR (near infra‐red) spectra (Ortiz et al., 2013). The forward PCA first calculates the eigenvectors, eigenvalues, singular values, and the square roots of the eigenvalues. These are then transformed by a varimax rotation which preserves the orthogonality of the eigenvectors (Judice et al., 2020; Kaiser, 1958). The varimax rotation simplifies the scaled eigenvectors (which are multiplied by their associated singular values and are thus referred to as component loadings) by finding the independent or orthogonal solution with the greatest separation between the coefficients of the large and small loadings (Kaiser, 1958; Ortiz et al., 2019).

The remote sensing analysis was a multi‐image VPCA applied to a time series of Sentinel‐2 MSI images using custom Python API scripts in Google Earth Engine. The multi‐image VPCA uses a spatially weighted average correlation matrix to derive six average independent component loadings from the image collection. These loadings were used to calculate component scores by fitting each pixel's derivative spectrum to each component loading and calculating least square coefficients. A positive component score constitutes a good fit between the derivative spectra, and a negative component score constitutes a poor fit. By projecting these component scores onto every image over time it produces a time series of component scores that capture spatial and temporal variability across the IRL from the beginning of 2020 to the end of 2022. The resulting component score maps were used to assess changes in water quality and identify the distribution of suspended sediment and macroalgae, and the dominant microalgae divisions in harmful algal blooms (HABs) present. When referring to the component spatially and spectrally for interpretation and identification it will be referred to as simply component 1–6.

The multi‐image VPCA follows this workflow; preprocess (isolate water pixels and calculate center wavelength derivative spectra) → calculate correlation matrix for each image → calculate weighted average correlation matrix by water pixels in each image → conduct varimax rotation and principal component analysis → compare component loadings to spectral library → compute component scores on all images → time series analysis and interpretation.

By employing all the bands in the VNIR, the VPCA method enables the application of VNIR derivative spectroscopy to the spectrally unmixed signatures, enabling another valid dimension of identification or attribution in the spectral domain rather than sole reliance on identification in the spatial domain (Vandermeulen et al., 2020). Each component loading generated by the VPCA is a spectral vector with a spectral shape that allows comparison to a spectral library for identification. To interpret the VPCA results it is critical to identify with great confidence what the components loadings represent. To achieve this in the spectral domain, we used a multifaceted statistical approach to calculate the best match using stepwise principal component multiple linear regression against a spectral library to determine which constituents are incorporated into each component. The principal component regression employs dual stopping criteria based on the significance of the *F*‐test and a variance inflation factor <2.00 for all coefficients in the model. This approach is applied to reduce the chance of overfitting. We then assessed the spatial fit and model validity using field collected sonde data, hyperspectral reflectance, and continuous field station data. This approach synthesizes data at three spatial, temporal, and spectral resolutions, employing hyperspectral field data, and multispectral remote sensing data from the harmonized Sentinel‐2 A/B MSI, and Sentinel‐3 A/B OLCI. The combination of spectral fitting along with empirical validation in the spatial domain allows for the attribution of the best match or matches to provide identification of components. This is in accordance with precedent set in papers like Judice et al. (2020), Rast and Painter (2019), and Avouris and Ortiz (2019). For identification of mixtures or generalized identification, the dominant contributing factors will be identified. Since the VPCA method uses derivative *R*_rs_, this makes it less sensitive to atmospheric errors and removes scattering biases (Ortiz et al., 2017). In addition, our spectral decomposition is based on the correlation matrix, and not the covariance matrix, which removes dimension or unit biases. And finally, the eigenvalue‐eigenvector decomposition isolates signatures and thus removes residual atmospheric bias (Ortiz et al., 2019). The VPCA is insensitive to the atmospheric correction used due to the spectral decomposition of the correlation matrix and derivative spectra rather than the absolute reflectance (Ortiz et al., 2017). This means that this method can be used with operational atmospheric corrections applied to standard Level 2 products (Judice et al., 2020.) and allows cross comparison of loadings from sensors with varying spatial, spectral, and temporal resolutions.

The approach accurately represents the constituents in the waterbody and was validated by comparison to in situ measurements and results from Judice et al. (2020). Judice et al. (2020) applies a similar methodology applying a VPCA analysis to level 2 surface reflectance data collected from Sentinel‐3A images after calculating the derivative spectra. One of the primary differences in methodology is that they identify components across 12 days using individually processed images instead of our average correlation matrix method for cross comparison. Sentinel‐2 A/B MSI and Sentinel‐3A/B OLCI sensors have significant differences that could affect interpretation of data, which is why it is important to validate using both. The most significant differences between the two satellite sensors are their spectral density, temporal, and spatial resolution. In this way, more independent information can be extracted from a data set than when using other remote sensing analysis methods. For Sentinel‐2 MSI, implementation of the averaging code now allows calculation of the component loadings based on the weighted average of multiple images.

### 2.2 Sentinel‐2 Data Processing

For Sentinel 2 data processing, the harmonized level 2 surface reflectance data set available in the GEE data catalog is used. This harmonization shifts the spectral response of the Sentinel‐2 B MSI sensor to match that of Sentinel‐2 A MSI spectral response (https://developers.google.com/earth‐engine/datasets/catalog/COPERNICUS_S2_SR_HARMONIZED). This data set has the Sen2Cor atmospheric correction algorithm applied to it. Sentinel‐2 A/B MSI has five bands in the visible between 400 and 700 nm compared to Sentinel‐3 A/B's 11 high SNR bands in the visible. The smaller number of bands and broader spectral widths of the Sentinel‐2 A/B MSI sensors make it more challenging to identify suspended sediment and pigments despite the finer spatial resolution. The visible portion of the EM spectrum (400–700 nm) is the critical range for identifying the primary response for pigment‐related signals from algae and vegetation. Sentinel‐2 and Sentinel‐3 have adequate spectral bands to allow detection of harmful algae due to strong response in the near infra‐red (Rodríguez‐Benito et al., 2020). Sentinel‐2 high spatial resolution and bands have been successful in extracting the spatial distribution of algae blooms with limitations in temporal frequency (Xu et al., 2021). The VPCA method has also been documented to increase the signal‐to‐noise ratio for remote sensing image analysis by up to 19x greater than the standard hardware SNR for a given sensor (Ortiz et al., 2019). As such, VPCA works much more effectively in low biomass conditions than traditional remote sensing methods (Judice et al., 2020). To account for the Sentinel‐2 A/B MSI sensors' coarse spectral and temporal resolution a large data set was used for the analysis incorporating 109 images into the average correlation matrix.

The multi‐image VPCA on Sentinel‐2 A/B MSI images is applied using custom Python spectral decomposition code optimized for use in the GEE COLAB API environment. The permalink is provided in the Open research section for access to the code used to run this analysis. The Sentinel‐2 A/B MSI sensors were selected due to the fine spatial resolution of its bands, which range between 10, 20, and 60 m depending on their spectral position. Bands near the ultraviolet and near infra‐red are at 60 m resolution. The coastal blue band (band1), which is a 60 m band, is utilized to extend the spectral range and assist in component identification. This analysis used Sentinel‐2 A/B MSI bands 1 (center wavelength 443.9 nm), 2 (496.6 nm), 3 (560.0 nm), 4 (664.5 nm), 5 (703.9 nm), 6 (740.2 nm), 7 (782.5 nm), 8 (835.1 nm), and 8a (864.8 nm), resampled to 10 m resolution.

Images are filtered using a 10% cloud coverage threshold and visually filtered for sun glint, poor atmospheric correction, and sensor error by visual inspection. The rejection criteria were widespread specular reflection, washed out water pixels, visible striping or sensor artifacts, and anomalous atmospheric correction behavior. If images exhibited any of these, they were not considered for inclusion. The 10% cloud cover is used to constrain primarily clear days and mitigate contribution of shadowing and cloud correction error. This is a conservative criteria due to the abundance of images during our study period. The Python code in GEE extracts water pixels from nine Sentinel‐2 A/B bands in the full image cube and applies the GEE QA60 (0) cloud mask and SCL (6) water mask to filter out cloud and land pixels. This enables analysis of only the water pixels in the image. It then calculates the derivative spectra and applies a three‐pixel kernel radius gaussian smoother to reduce isolated pixel scale variability and residual noise. For each image within the data set a correlation matrix is calculated. They are then combined to obtain a weighted‐average correlation matrix.

In previous work using Sentinel‐3 A/B OLCI, separate correlation matrices were calculated for each image using the ENVI/IDL software package. The component loadings from each individual image were matched by comparison with a spectral library (Judice et al., 2020). Having demonstrated that the same component identification could be pulled from multiple images, an average image was created from 11 images across the seasonal cycle to obtain an average correlation matrix. The average correlation matrix was then used to create a time series from the average components by projecting them on to the individual images (Judice et al., 2020). To improve that approach this study synthesizes over 109 images into the same weighted‐average correlation matrix as input for the VPCA decomposition. This gives a more robust analysis of present constituents over time. The average correlation matrix and VPCA analysis on Sentinel‐2 A/B MSI through seasonal cycles allows for creation of seamless GIF animations to reveal patterns of variability throughout the year. This visualizes the development of component score patterns such as algae blooms, seagrass extent, and suspended mineral plumes throughout the year.

### 2.3 Sentinel‐3 VPCA Spectral Decomposition

For the purposes of this study Sentinel‐3 A/B OLCI data was processed in the same procedure as the Sentinel‐2 MSI data shown in Section 2.1. This was a single image analysis, so we did not create an average correlation matrix before conducting the VPCA. The clearest Sentinel‐3A OLCI image from the field sampling days (8/3/22) was selected and downloaded from EUMETSAT Copernicus data browser. Sentinel‐3A OLCI level 2 product water pixels are used for the analysis. These pixels have a 300 × 300 m resolution. Bands Oa1 (center wavelength 400 nm), Oa2 (412.5 nm), Oa3 (422.5 nm), Oa5 (510.0 nm), Oa6 (560.0 nm), Oa7 (620.0 nm), Oa8 (665.0), Oa9 (673.8 nm), Oa10 (681.3 nm), and Oa11 (708.8 nm) were used for the analysis. The Harris Geospatial ENVI/IDL software was used to calculate the center‐weighted derivative for Sentinel‐3A OLCI's 11 visible light bands. VPCA decomposition was conducted on the image using the ENVI/IDL software. Six components were extracted in this analysis to match the Sentinel‐2 MSI analysis. The 8/3/22 Sentinel‐3 scene was selected because it is coincident with Sentinel‐2 and field sampling. This provides a coincident cross‐sensor comparison at greater spectral resolution but coarser spatial resolution while also enabling comparison to field collected hyperspectral data.

### 2.4 Field Data and Kilroy Data Processing

Field data was collected on 2 days on August 1st and August 3rd in 2022. These dates were selected for their coincident flyovers of Sentinel‐2 MSI. At each station we collected date, time, GPS location, water depth, and MET conditions. GPS coordinates were collected using a smartphone. We took photos of the water, and sky at the site, conducted a Secchi measurement, and a Yellow Springs Instrument (YSI) sonde profile. We also collected water samples in brown 125 ml HDPE bottles. These samples were collected from the surface water, preserved, and later analyzed for enumeration, biovolume (BV) estimation, followed by cell count analysis, which identified cells to the Genus and Division level.

Sediment was collected from the lagoon bottom at each site on 8/3/22. These samples were collected by lowering an Ekman dredge. This sediment was then transferred to sediment sample containers, which were kept on ice, partitioned and frozen in the laboratory. These samples were then freeze dried and ball milled to homogenize them before being analyzed using an Elemental Analyzer to collect CaCO_3_ wt.%, total carbon wt.%, total organic carbon (TOC) wt.%, total inorganic carbon wt.%, total nitrogen (TN) wt.%, and carbon: nitrogen (C:N) ratios wt.%/wt.%.

Secchi depths were collected using a standard 8‐inch, black and white quadrant Secchi disc. This measurement represents the clarity of the water column and can therefore point toward the level of turbidity within the area. A YSI EXO field sonde collected measurements of turbidity, Chlorophyll‐a, conductivity, depth, fluorescent dissolved organic matter, Conductivity, salinity, optical dissolved oxygen, total suspended solid, total algae (TAL), and temperature. Yellow Springs Instrument sonde data was obtained from each sample site from the entire water column collecting continuous data on descent and ascent. Photosynthetically active radiation (PAR, 400–700 nm) is equivalent to the optical depth of the Secchi depth and was important because it determines the conversion between Secchi depth and photic depth, conventionally, the depth of penetration of 1% of surface irradiance (Gallegos et al., 2011). We took the average of each sonde data sample listed above at each field site from the surface to one Secchi depth as an approximation of the water column that is predominantly influencing the satellite remote sensing signal. This data could then be used to determine correlation with component scores.

Field spectral data was collected using a Malvern Panalytical (formerly ASD) FieldSpec Handheld 2 (HH2) hyperspectral radiometer with a 10° fore optic and RCR cosine theta receptor. The instrument measures in the visible/near infrared wavelength range from 400 to 900 nm. The reflectance data is initially reported at 1 nm hyperspectral resolution. However, the instrument has a measurement resolution of ∼5 nm in the visible and ∼10 nm in NIR. The samples were thus interpolated to 10 nm resolution for hyperspectral analysis and derivative transformation. The results were also resampled to Sentinel‐2 resolution to evaluate the effect of spectral density on identification of component loadings at different resolutions. The interpolation to Sentinel‐2 resolution includes averaging over the bandwidths of Sentinel‐2 bands 1 through 8a and convolution with the Sentinel 2 band response functions before running the VPCA to best mimic how Sentinel‐2 images are processed. This data loss from interpolation is represented in Figure S1 in Supporting Information S1. For the field data, the derivative spectra were compiled into a matrix that can be imported into the SPSS Statistics™ software to apply the VPCA decomposition method.

Kilroy hyperspectral data was collected from Kars Park, Barge Junction, and Grand South using paired Ocean Optics spectroradiometers. One of the two spectrometers was downward looking and equipped with a 10° fore optic, while the other was upward looking and equipped with a cosine theta RCR receptor to measure downwelling irradiance. The instruments were intercalibrated in the lab. The ratio of the upwelling radiance divided by the downwelling irradiance yields total remote sensing reflectance in sr^−1^. Twenty‐one spectra were selected from April to December of 2022. These spectra were visually inspected to ensure they are valid spectra reflectance and not influenced by glare. The hyperspectral data was interpolated to 10 nm and the derivatives were calculated. The derivative spectra were compiled into a matrix that can be imported into the SPSS software to apply a VPCA decomposition method.

### 2.5 Comparison to Spectral Library

Stepwise principal component multiple linear regression was used to compare the component loadings calculated in the VPCA to a spectral library. A USGS standard mineral and pigment spectral library compiled with other independently published spectral libraries was used to identify constituents (Avouris & Ortiz, 2019; Ortiz et al., 2019). The spectral library was refined to include spectra of constituents that were likely to be present in the IRL. Standard regression statistics *r*, *F*‐value, and *p*‐value were assessed along with variance inflation factors (VIF) to test the level of multicollinearity. The VPCA loadings were fit to as many matching spectral constituents as were *p* < 0.05 and with VIF < 2 for each individual coefficient. This VIF threshold has been used in previous studies to minimize the risk of overfitting (Judice et al., 2020; Ortiz et al., 2019). This VIF threshold is a conservative criteria to limit collinearity in library predictors. The library for comparison was interpolated at 10 nm hyperspectral, Sentinel‐3A OLCI, and Sentinel 2 A MSI resolution so that they can be compared to the component loadings from each data set respectively. The data within the library consists of pigment related spectra as well as mineral related spectra. Since many pigment products do not measure outside of the visible spectrum while minerals do, the mineral spectra were truncated to match pigments at 709 nm for Sentinel‐3, 703.9 nm for Sentinel‐2 and 700 nm for the 10 nm interpolated hyperspectral data sets. For further analysis and validation, the 2020 to the end of 2022 component analysis was conducted in three ways: using both pigment and mineral spectra from 400 to ∼700 nm, with pigment and pigment degradation products only, and with minerals only out to 864.8 nm. The results from each stepwise principal component multiple linear regression were compared for consistency in identification and attribution.

### 2.6 GIS Pixel Extraction and Map Generation

To further validate the 2020–2022 average Sentinel‐2 MSI (multi‐image Sentinel‐2) component identifications, the Quantum Geographic Information System (QGIS) software program was used to analyze the component score maps produced by the VPCA decomposition. QGIS was used to map the component scores and to extract the pixel values at the coordinates of the water sampling stations. Histograms were calculated to determine a proper range of values of the component scores to maximize the map interpretability. The colors applied to the component scores represent the strength and direction of the variance for each component at each pixel, relative to the mean of the original data. These are plotted with positive values (warm colors) indicating higher association of the pixels with the spectral constituent mixture represented by this component while negative values (cool colors) indicate a lower association. The extracted pixel values were then compared to the sonde data averaged over the Secchi depth and a stepwise principal component multiple linear regression was conducted against each component loading to identify the constituents present.

## 3 Data Results

### 3.1 Field Cell Count, BV, and Sonde Data

Figure 2 represents the Division of algae by biovolume collected from the water samples. All locations were heavily dominated by Miozoa except for Barge Junction that recorded dominance of bacillariophytes on the first sample day and cryptophytes on the second day. Barge Junction also recorded lower BV compared to other locations. When comparing the Miozoa BV to total BV we see a strong correlation but not when comparing Miozoa to cell count. This is due to the dinoflagellate cells being larger than the cyanobacteria cells and their relative abundances shifting between stations. The biomass present reaches up to 3.02E+10 μm^3^/L at Sykes Creek on 8/1/2022 and 3.60E+10 μm^3^/L at the Grant South site on 8/3/2022. Miozoa Cells/L count ranged from 6.01E+04 at Kars Park on 8/3/2022 to 7.38E+05 Cells/L at Grand south on 8/1/2022. These values are consistent with bloom conditions as determined by Stumpf et al. (2022) that defined K. Brevis dinoflagellate bloom presence as >5,000 Cells/L and this corresponds to the regulatory threshold in Florida water management for shellfish harvesting. During field sampling all cell count samples at all locations would be considered bloom presence under these criteria when compared to Miozoa.

**Figure 2:**
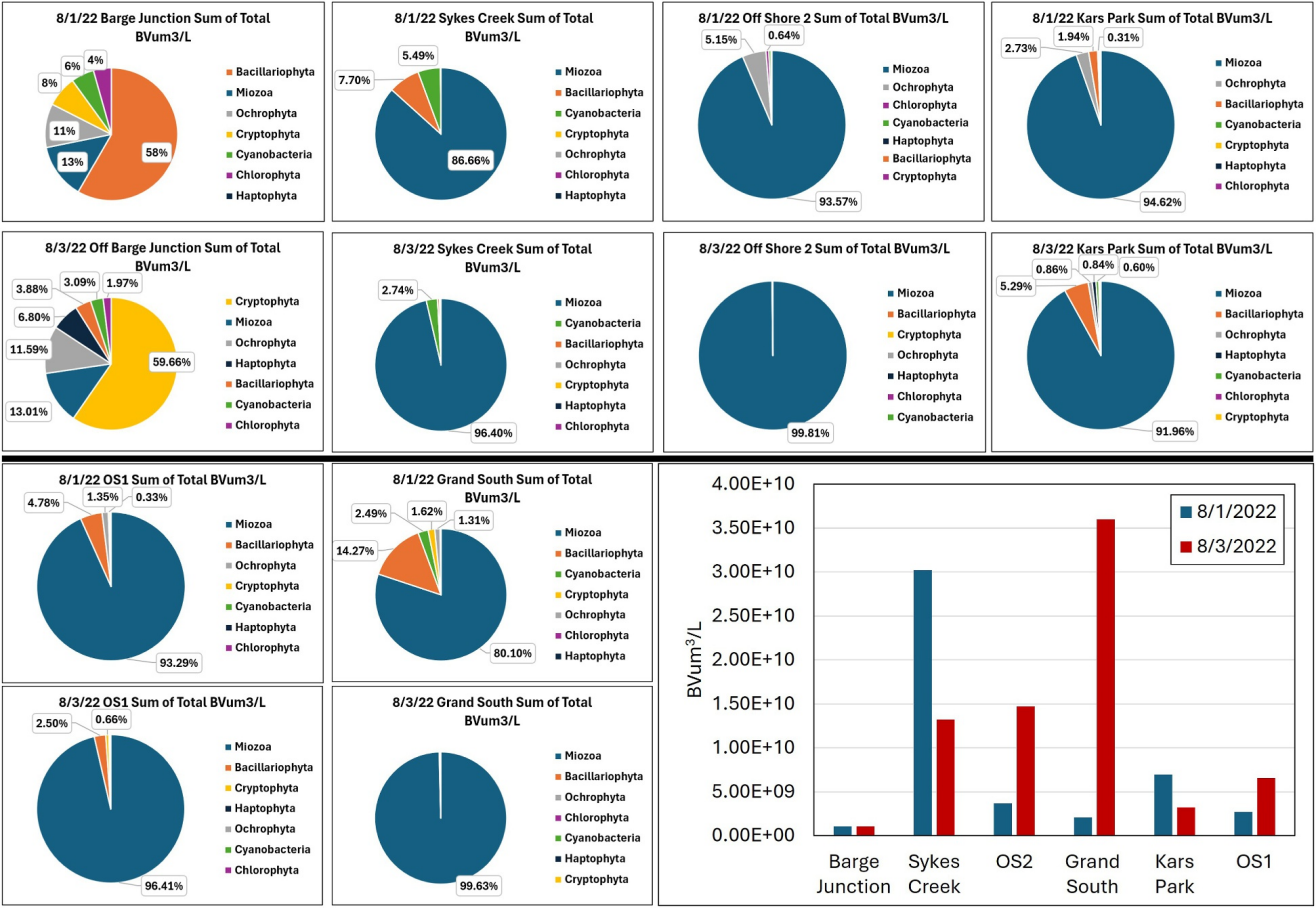
Pie charts showing the percentage of each Division of Algae by biovolume (BVum^3^/L) at each sampling location. The top row represents the first day of sampling on 8/1/22 with the following field day 8/3/22 shown directly below it. A bar chart showing the quantity of biovolume (BVum^3^/L) is shown in the bottom right.

Using multi‐image Sentinel‐2 component score maps compared 8/3/22 sonde collected data to extracted component pixel values. Multi‐image Sentinel‐2 Components 1, 2, and 6 exhibit strong negative correlation to Chl ug/L. Multi‐image Sentinel‐2 Components 3, 4, and 5 exhibit strong positive correlations to Chl ug/L. This indicates that on 8‐3‐22 suspended sediment and cyanobacterial related components are negatively correlated with Chl‐a while dinoflagellate and seagrass were positively correlated. This is consistent with the observed dominance of dinoflagellates during field sampling. These correlation scatterplots can be found in the Figure S14 in Supporting Information S1.

### 3.2 Sentinel‐2 Averaged Component Loadings

The spectral products of the multi‐image VPCA analysis are component loadings which represent the derivative spectral shape of the most prominent reflectance signals throughout the 3‐year period. By calculating these component loadings and matching them to a spectral library, it is possible to identify the source of the water quality changes through time.

Figure 3 represents the component's eigenvalues and fractional variance of each component. The fractional variance for each component rank represents the amount of variance that each component explains. The VPCA model unmixes the spectral data collected in each pixel of the Sentinel‐2 image. The analysis accounts for 96.7% of the variance in the images and unmixed Component 1 represents 33%, Component 2 represents 24.8%, Component 3 represents 23.3%, Component 4 represents 6.8%, Component 5 represents 4.6%, and Component 6 represents 4.2%. The six components were retained due to meeting the following criteria. The first six components exceeded a total variance threshold of 95% that is commonly applied for component selection. This indicates that most of the variability within the data set is captured within these six components. Second, we inspected the scree plot showing a clear diminishing return after the 6th component with those residual components contributing only marginal variance. Lastly the six component spectral fingerprints were spectrally matched and supported by field observations and previously published observations, whereas higher order components primarily represent noise or minor residual variability. Although component 5 and 6 account for a small portion of variance, and small difference in variance, they were retained due to their independently interpretable loading spectra and spatial score patterns that were supported by field observations and previously published observations. Future applications of the multi‐image VPCA could incorporate bootstrap resampling to quantify uncertainty which would provide added robustness to the component selection criteria.

**Figure 3:**
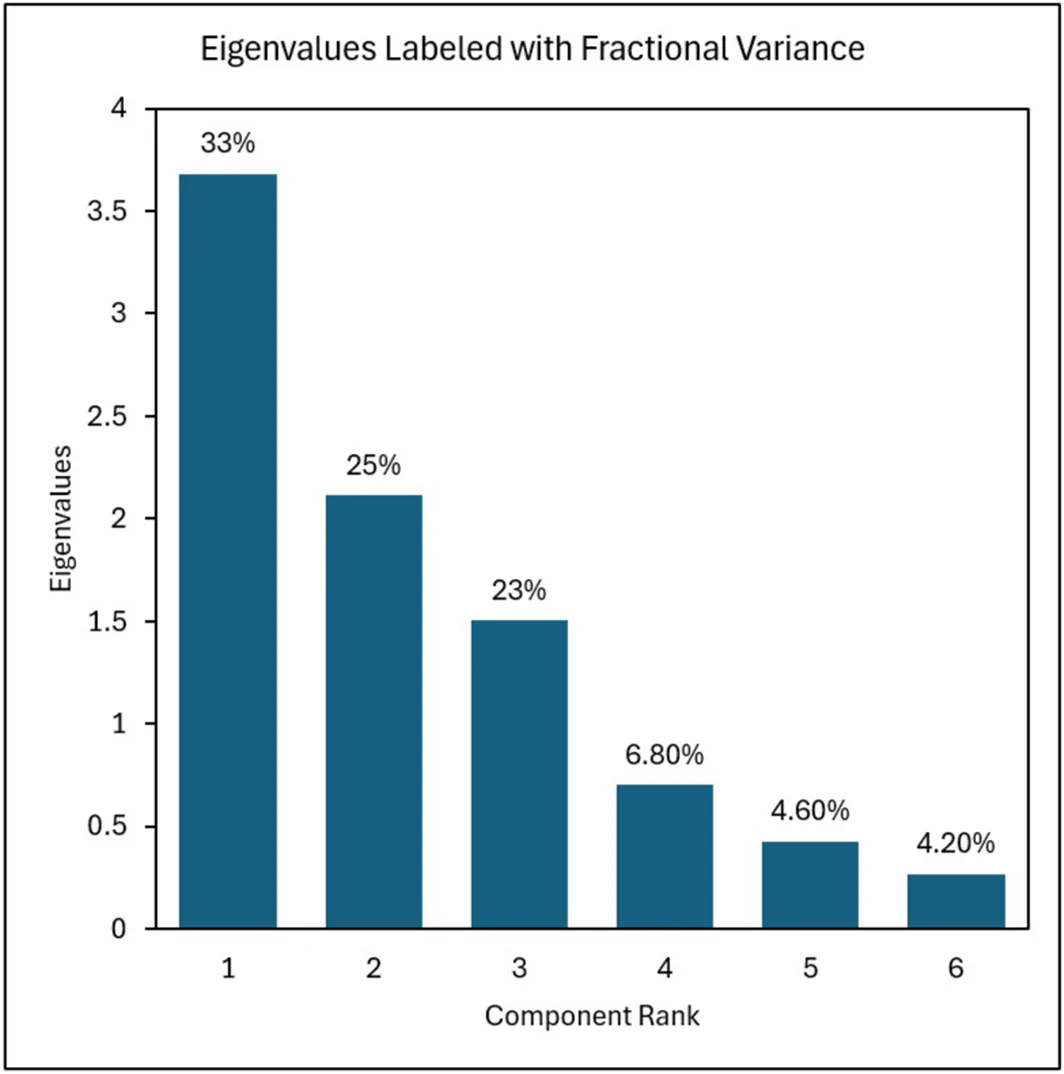
This bar chart shows eigenvalues for each component rank. This shows the amount of variance each component represents.

Figure 4 represents the comparison of the multi‐image VPCA component loading *Z* scores. The component *z* scores are calculated from the component loadings and library spectra in order to standardize the value over its distribution for comparison. The sign of VPCA components is mathematically indeterminate due to the nature of the varimax rotation process. The VPCA loading spectra for Component 1 and 6 were inverted relative to reference spectra for simplicity of interpretation in Figure 4.

**Figure 4:**
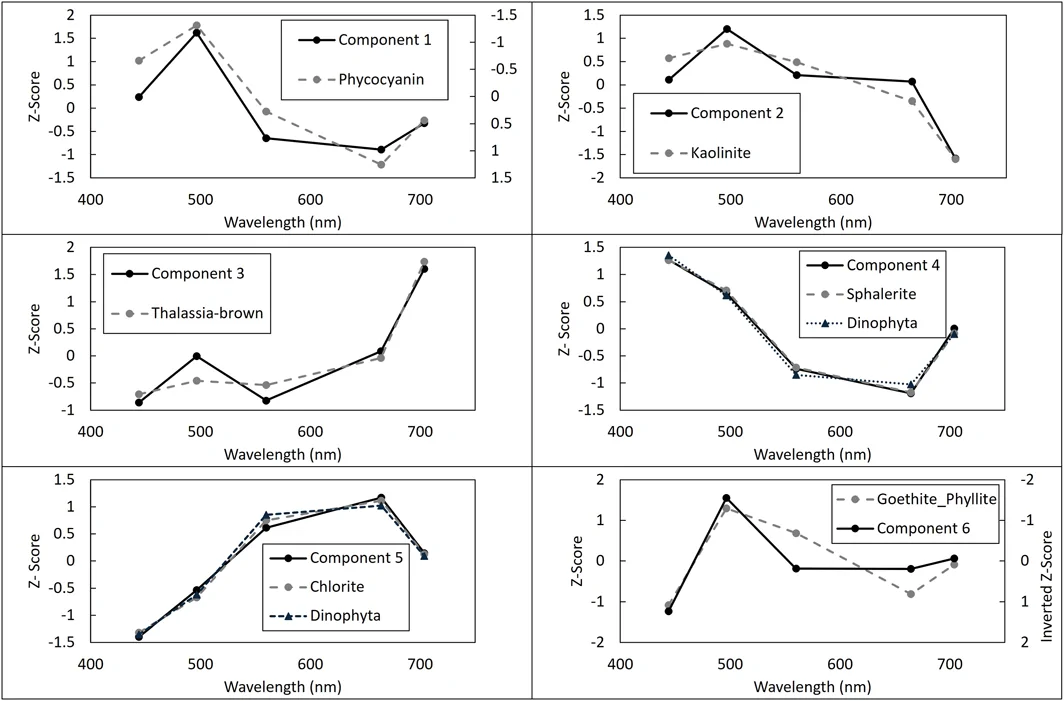
Sentinel‐2 multi‐image varimax‐rotated principal component analysis component standardize spectral loading *Z* scores compared to best match spectral library *z* scores. Component 1 *Z*‐score matches phycocyanin (*r* ^2^ = 0.865; *p*‐value = 0.02). Component 2 *Z*‐score matches kaolinite (*r* ^2^ = 0.865; *p*‐value = 0.02). Component 3 *Z*‐score matches Thalassia‐brown (*r* ^2^ = 0.916; *p*‐value = 0.01). Component 4 *Z*‐score matches sphalerite (*r* ^2^ = 0.996; *p*‐value = 0.0001) and dinophyta (*r* ^2^ = 0.986; *p*‐value = 0.0007). Component 5 *Z*‐score matches chlorite (*r* ^2^ = 0.989; *p*‐value = 0.0006) and dinophyte (*r* ^2^ = 0.978; *p*‐value = 0.001). Component 6 *Z*‐score matches goethite (*r* ^2^ = 0.711; *p*‐value = 0.07).

### 3.3 Sentinel‐3 VPCA Decomposition on 8/3/22

To validate the Sentinel‐2 multi‐image VPCA, a single Sentinel‐3 image, collected on 8/3/22 to match the field sampling and Sentinel‐2 pass over time, was collected and analyzed by VPCA in ENVI/IDL. Six components were extracted to match the analysis using Sentinel‐2 on the same day. We calculated and identified the component loadings and represented them in the summary plots in Figure 5. The component graphs show the spectral shape of the component loadings and the component score maps to represent the spatial extent of the components.

**Figure 5:**
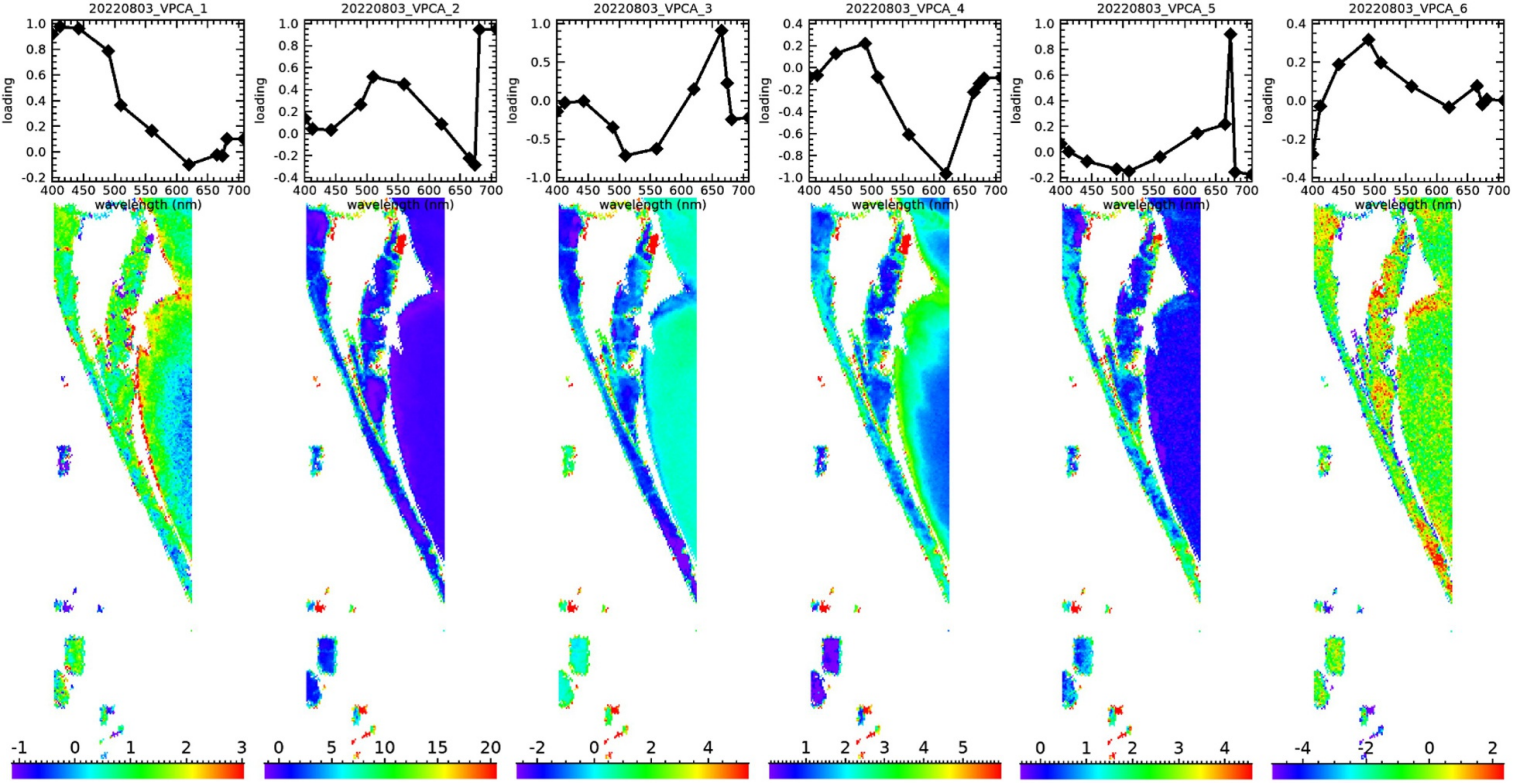
Sentinel‐3 OLCI varimax‐rotated principal component analysis analysis using the same methods used by Judice et al. (2020) to identify brown tide. This analysis represents the 6 leading components using Sentinel‐3. The spatial maps represent component score maps. Warmer colors indicate higher association of the pixels with the spectral constituents represented by this component while cool colors indicate a lower association.

### 3.4 Component Identification

Five separate VPCA analyses were generated on different data sets for this study. Nearly all component loadings matched a constituent in the spectral library. The library has been developed as a compilation of spectra taken from published sources and compiled to match what is likely to be in the lagoon based on prior work. For example, *Thalassia* green (healthy) and brown (unhealthy) were added due to a known population of sea grass in the IRL. There are 67 minerals and 68 pigments or algae spectra included in the version of the spectral library used here.

Figure 6 represents the calculated component loading identifications across data sets and sensors. Component loadings that are attributed to the same water components are color coded and labeled at the top. The component loadings extracted from the five different VPCA models vary somewhat due to spectral, spatial and temporal differences between the data sets. Despite this as shown in Figure 6, similar component loading IDs are observed within the various data sets. Figure 4 shows the spectral graphs of the multi‐image VPCA for Senitinel‐2 along with the best matches within the spectral library. All the component loadings had one spectral match except for Component 4 and 5 that exhibited ambiguities between dinoflagellate and sediment constituents. Multi‐image Sentinel‐2 MSI Component 1 was identified as Phycocyanin (Cyanobacteria) due to a spectral match of *r* ^2^ = 0.865, and validation by 10 nm field ASD, Sentinel‐3 8/3/22, and 10 nm Optical Kilroy component analysis. Multi‐image Sentinel‐2 Component 2 was identified as kaolinite with a spectral match of *r* ^2^ = 0.865 and validated by 10 nm ASD field data and Sentinel‐2 single image VPCA with minerals only. Multi‐image Sentinel‐2 Component 3 was identified as *Thalassia*‐brown representing unhealthy seagrass with a spectral match of (*r* ^2^ = 0.916) and was validated by 10 nm ASD data as well as reported seagrass bed maps. It was supported by consistent Sentinel‐2 single day Component 1 analysis as Sargassum, which is a form of seaweed. Multi‐image Component 4 was a spectral match to Sphalerite (*r* ^2^ = 0.996) and was validated by 10 nm field data, Sentinel‐3 MSI analysis, and 10 nm Optical Kilroy. Multi‐image Sentinel‐2 Component 5 was a spectral match to Chlorite (*r* ^2^ = 0.989) and was validated by the field ASD samples, single image sentinel‐2 VPCA, and the Kilroy samples. Multi‐image Sentinel‐2 Component 6 was identified as Goethite with a spectral match of *r* ^2^ = 0.711 when the probability of F was reduced to 0.09. This identification was validated by the field ASD and the Optical Kilroy data. In a pigment only analysis multi‐image Sentinel‐2 Components 4 and 5 are attributed to Dinophytes with a *r* ^2^ = 0.986 and *r* ^2^ = 0.978 respectively which are slightly less significant than the sphalerite and chlorite signal attributed to those two components. However, due to the preponderance of evidence from field observations at this time, Dinophyta and/or iron sulfate mixtures are identified as the most likely dominant contributing factor for Component 4 while Component 5's dominant contributing factor is Dinophyte and/or chlorite mixture with possible greater contribution after high turbidity events. Its presence was also supported by appearance in the spectral field data, observations in Phlips et al. (2021) biological field cell count study, Sentinel‐2 single day analysis, Kilroy station data, and cell count and BV analysis from this study presented in Figure 2.

**Figure 6:**
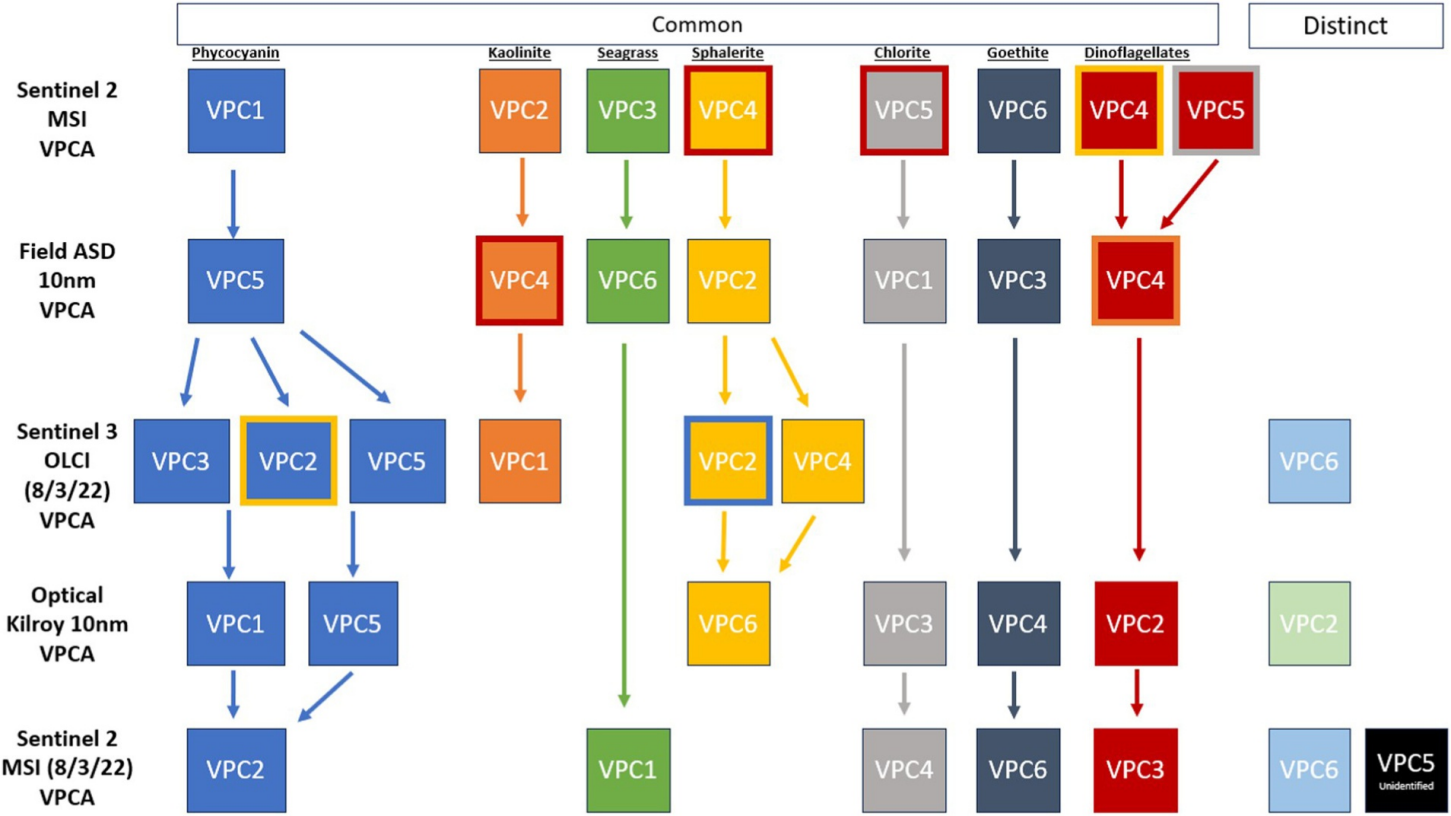
Component loading identification matched with similar constituents between Sentinel‐2 A/B MSI average varimax‐rotated principal component analysis (VPCA), Field collected 10 nm resolution VPCA, Sentinel‐3 VPCA 8/3/22, Optical Kilroy 10 nm resolution VPCA, and Sentinel‐2 A/B Single Image VPCA 8/3/22.

### 3.5 Seagrass Identification

Figure 7 represents a comparison of the seagrass related component score map to a seagrass study conducted in 2017. The image was selected on a day with low turbidity to best represent the seagrass related component. There are small differences that can be attributed to changes in area coverage between the survey in 2017 and the component analysis in 2020. Since these collections are 3 years apart, they are intended to demonstrate a strong spatial correspondence rather than an accuracy assessment. This shows remarkable agreement and validation for the Sentinel‐2 multi‐image VPCA analysis and its ability to identify seagrass beds.

**Figure 7:**
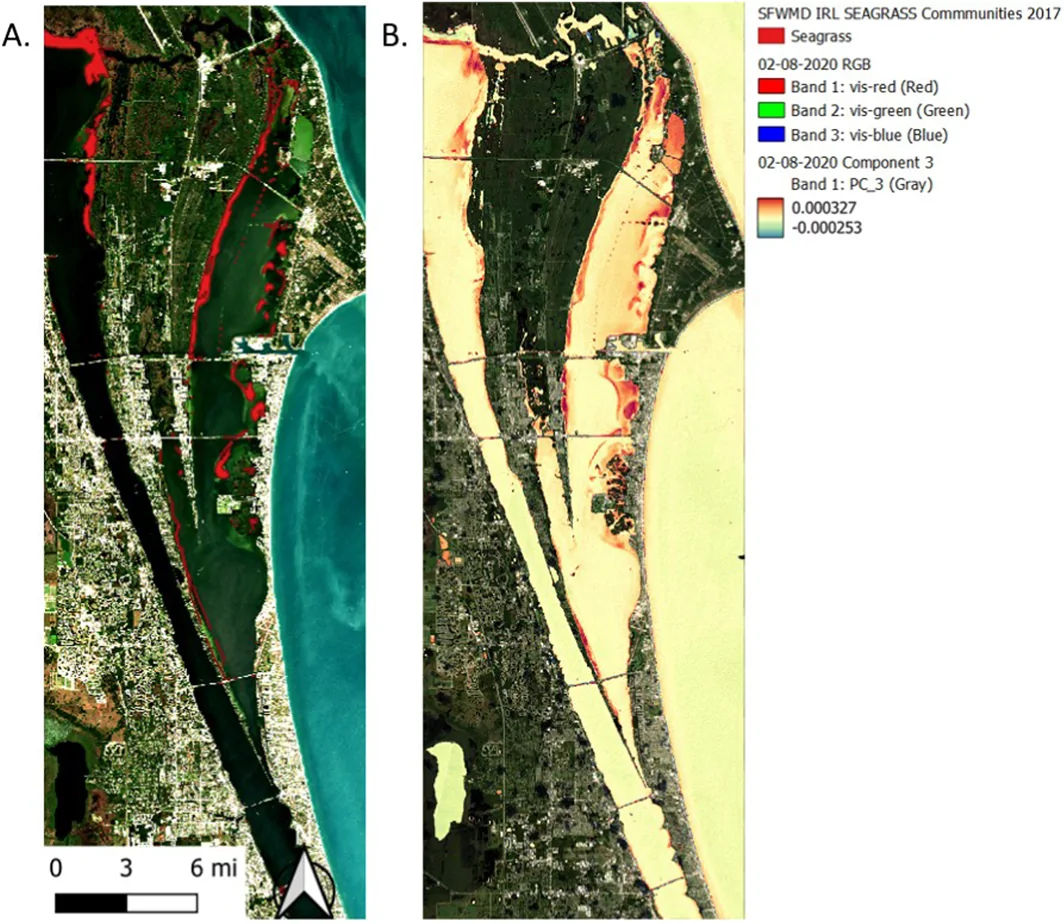
Spatial comparison of Seagrass polygons from South Florida Water Management District seagrass survey over Sentinel‐2 RGB image on 2/8/2020 (panel a) to varimax‐rotated principal component analysis seagrass related component score map extracted from Sentinel 2 A/B MSI average 2020–2022 on 8/3/22 (panel b). Warmer colors in panel (b) indicate higher association with the seagrass component while cool colors indicate a lower association.

### 3.6 Tracking HAB Development

Figure 8 represents a reported major cyanobacterial bloom that developed and collapsed at the end of 2020 (Phlips et al., 2021). This observation was able to be represented as a time series due to the multi‐image VPCA applying the phycocyanin related component 1 score to all images across the 2020 to end of 2022 period. Optical inspection for potential blooms was performed by inspecting gifs of component score maps and then validated by ORCA field station data. The ORCA Kilroy monitoring data indicated high blue green algae concentration based on phycocyanin sensing along with high Chl‐a supporting that Figure 8 is consistent with the reported cyanobacterial bloom.

**Figure 8:**
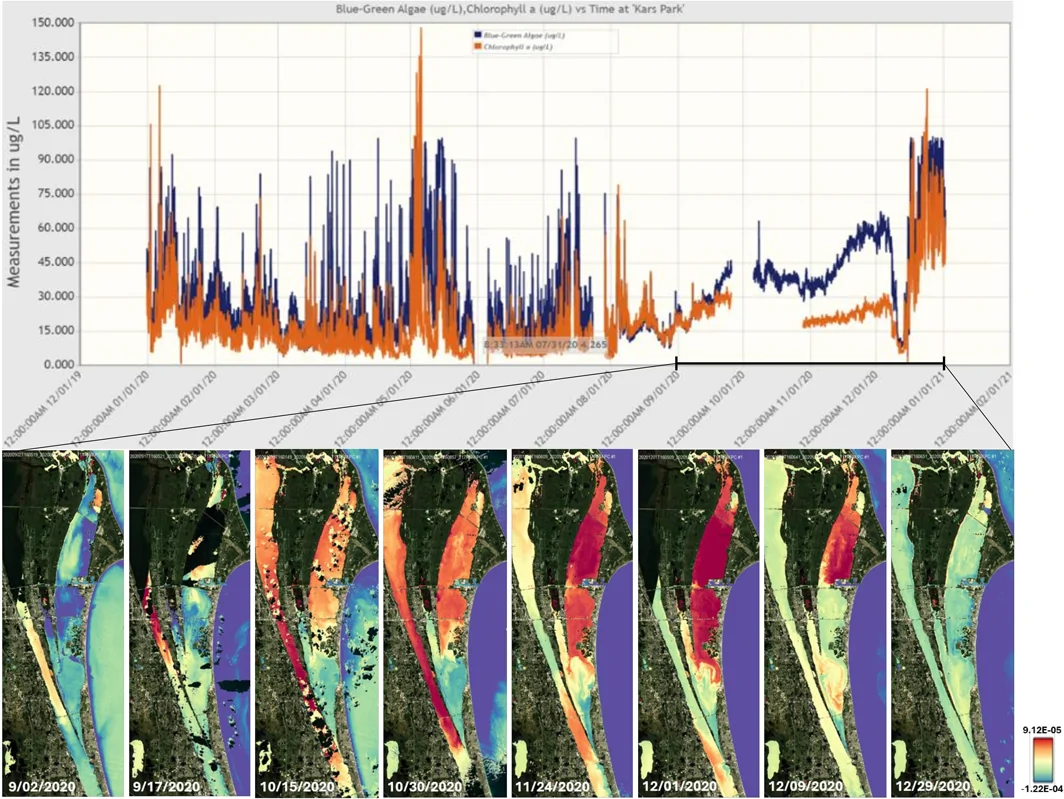
2020 Cyanobacterial bloom development and collapse reported in Phlips et al., 2021 observed in Component 1 score map (bottom panel) and supported by heightened blue‐green algae in relation to chlorophyl measured by Kilroy Kars Park Field station (top panel). Warmer colors indicate higher association of the pixels with the cyanobacterial component while cool colors indicate a lower association.

## 4 Discussion

### 4.1 Ambiguities of Utilizing Sentinel‐2 A/B MSI

The Sentinel‐2 A/B MSI sensors are powerful remote sensing tools for identifying water quality constituents; however, they are not without limitations. While the Sentinel‐2 A/B MSI sensors do provide exceptional spatial resolution to 10 m, the increase in spatial resolution comes at the expense of spectral density. This makes component identifications for certain materials more challenging. Studies into the spectral response of pigments do not typically extend into the NIR, thus the additional NIR sensor bands of the MSI sensor do not help to differentiate pigments, although they can assist with the identification of minerals. One might expect to observe differences in identification between various data sets due to different spectral, temporal, spatial resolutions, and data set sizes. Despite this, there is remarkable consistency in spectral identification when comparing across data sets and sensors used in this study shown in Figure 2.

The limited spectral resolution is addressed by increasing the data set size to 109 images. With increased data set size, component decomposition can better differentiate small differences between signals with some generalization between component identifications. In this case when matching sediment minerology, it is reasonable to generalize suspended sediments. Despite Component 5 having possible matches to other minerals, Figure 4 shows a confident match to Dinophyta giving us an ability to differentiate between algae divisions. The Optical Kilroy, Field ASD data, and pixel extraction were used to guide the identification of average satellite VPCA components. By cross validating using sensors and collecting field data on the same day across satellites, field hyperspectral instruments, with varying spatial and spectral resolution we have further validated the limitations of the sensors band resolution, temporal frequency, and sun glint.

Sentinel‐2 MSI's revisit time is ∼5 days which is coarse compared to Sentinel‐3's daily revisit time. This limitation is made more prominent by periodic cloud cover that can further degrade temporal resolution. To create a robust and representative average correlation matrix a 10% cloud coverage filter was applied to every available image and from that subsection each image was optically analyzed and filtered for any evidence of sun glint, poor atmospheric correction, or sensor error. This yielded 109 images over a 3‐year period. By filtering in this way, it is possible to maintain the accuracy of the correlation matrix while reducing the influence of sensor error and sun glint. This robust correlation matrix can then be applied to every available image with <20% cloud coverage, increasing the number of days analyzed for trends.

To address ambiguities, several sensors were used for validation. Sentinel‐2 MSI has only five broad bands in the pigment response range of 400–700 nm which smooths the spectral response making it difficult to validate findings. To address this, several different resolutions from other sensors were utilized to help identify and validate the average Sentinel‐2 loadings. On the field sampling day 8/3/22 a single image from Sentinel‐2 MSI, handheld hyperspectral data at each of the six sampling sites, a Sentinel‐3A OLCI image, and Kilroy hyperspectral field sensors data (April to December) was collected. The VPCA method was applied to each data set independently, with their spatial, temporal, and spectral differences but yielded remarkably similar identifications and spatial patterns as shown in Figure 5. Although there were some ambiguities in the identification of clay minerals, if clays are grouped together, they show remarkable similarities across data sets. This, combined with a significant amount of data in 109 images incorporated into the 3‐year average correlation matrix, provides a more accurate representation of the constituents and allows great confidence in the identification and interpretation of the components yielded by the Sentinel‐2 averaging VPCA approach.

### 4.2 Confidence in Identification

To address the course band resolution in Sentinel‐2 MSI it was critical to identify spectra within the library that were similar. Once identifications were made there was a pattern of several different clay minerals with significantly similar spectra. With further analysis it became clear that clay mineral spectra were too similar to differentiate at the band resolution of Sentinel‐2. By broadening our identification criteria to group any clay mineral identified as a match, excellent agreement across sensors was achieved. Figure 6 shows the agreement of component identifications across sensors showing spectral matches across temporal, spectral, and spatial resolutions.

Multi‐image Sentinel‐2 Component 1 had excellent agreement across sensors but does not appear in the single image Sentinel‐2 MSI VPCA, likely because it was represented by Bchl‐a. Component 1 phycocyanin is assumed to be indicative of cyanobacteria in inland waters, but it is also present in Cryptophytes and Rhodophytes (Dring, 1992; Judice et al., 2020). Supporting evidence of Component 1, identified as phycocyanin, representing cyanobacteria is that the spatial patterns closely match reported in situ field and lab biological measurements during the year 2020 when a cyanobacterial bloom was recorded by multiple sources (Abbe et al., 2022; Phlips et al., 2021). For the analysis here multi‐image Sentinel‐2 Component 1 is confidently identified as cyanobacteria.

Multi‐image Sentinel‐2 Component 2, Kaolinite, was likely represented by Smectite in the Sentinel‐3 MSI component analysis due to it being a clay mineral with a similar response at this spectral resolution. The small differences in mineral spectra are better identified in the Sentinel‐2 average likely due to significantly larger data set sizes. Even so, due to the spectral signal of clay minerals at Sentinel‐2 MSI and Sentinel‐3 OLCI resolution being so similar we decided to group clay minerals together. Geochemical analysis on IRL sediments are consistent with the presence clay, carbonate, and phosphorite‐rich layers (Chevis et al., 2021). For further analysis multi‐image Sentinel‐2 Component 2 is confidently identified as suspended sediment related to clays.

For multi‐image Sentinel‐2 Component 3, the pattern of the seagrass beds was too spatially fine for Sentinel‐3OLCI's coarse spatial resolution, and the pixels were blended too much for it to be a primary component. This can be seen in the component maps in Figure 5. This combined with the significant temporal differences is likely why it appears in the Sentinel‐2 MSI analysis and not in Sentinel‐3 OLCI. Multi‐image Sentinel‐2 Component 3 shows remarkable similarities to reported seagrass beds shown in Figure 7. Multi‐image Sentinel‐2 Component 3 is confidently identified as seagrass.

For multi‐image Sentinel‐2 Component 4, it is ambiguous whether it represented iron bearing suspended sediment, dinoflagellates, or a mixture of the two. This is due to agreement across different sensors for the identified minerals as well as agreement with dinoflagellates. Multi‐image Sentinel‐2 Components 4 and 1 show remarkable agreement in spatial patterns with reported dinoflagellate bloom of *P. bahamense* in the summer followed by a fall bloom of nanoplanktonic cyanobacteria (Phlips et al., 2021). This along with prominence of Miozoa (dinoflagellates) in the sampled BV data makes it likely that the dinoflagellate signal was being hidden by the Sphalerite signal. The identification is also partially influenced by library bias and not having spectra for the specific dinoflagellate species seen in this bloom. Despite this, it shows agreement by matching *K. brevis* or Dinophyta in the spectral matching which is also supported with identification across sensors. We acknowledge that isolating the spectral reflectance of species level algae would be beneficial to continued research, however, there are few sources for species level reflectance. Sphalerite is a metallic mineral phase in Pb–Zn deposits, and has sink capacity for many trace elements, including primarily Fe, Mn, Cd, Ga, Ge, In, Hg, Ag, and Cu (Liu et al., 2022). Sphalerite can be formed in many ways including low to high temperature hydrothermal conditions; in coal, limestone, and other sedimentary deposits (GEOLOGYSCIENCE, 2019). In the IRL and the St. Lucie Estuary Copper (Cu) and zinc (Zn) transport after agricultural applications have accumulated in the sediments and are suspected to affect fish health (He et al., 2006). It is postulated that anomalously high concentrations of zinc, and sphalerite, are the result of precipitation from zinc‐ and lead‐bearing brines in a reducing sulfur‐rich environment (Swaine, 1994). These conditions are similar to the IRL setting. Sulfate reducing bacteria have a noted presence in the IRL (Bradshaw et al., 2020) and these are known to precipitate low solubility metal sulfides, with particular susceptibility to zinc inclusion in sulfide phases (Peltier et al., 2011). Iron is precipitated as Fe‐sulfide in an overlying OC‐rich sedimentary layer in the IRL (Roy et al., 2010). The precipitated Fe‐sulfides are remobilized at the sediment–water interface when oxygen‐rich lagoon water recirculates through the shallow sediments (Roy et al., 2010). Solid solution or exchange of Zn and Fe in iron sulfides precipitate at low temperature could yield a signal like the sphalerite that is observed. These dynamics observed in the IRL give some credit to the presence of Sphalerite, however, we believe further research is needed to determine if an average component could determine the suspended sediments minerology. Multi‐image Sentinel‐2 Component 4 is likely a precipitated iron sulfide formed through iron diagenesis and dinoflagellate associated mixture. When multi‐image Sentinel‐2 Components 4 and 5 are analyzed with only pigment products they both identify as Dinophyta (dinoflagellates).

Multi‐image Sentinel‐2 Component 5 is also closely associated to dinoflagellates and/or chlorite. While the spectral signatures of clay minerals and dinoflagellates are optically similar at Sentinel‐2 spectral resolution, the observed components likely represent mixed signals comprising varying mineral compositions in combination with dinoflagellates. This interpretation is further supported by the strong correlation observed between multi‐image Sentinel‐2 Components 3, 4, 5 and field‐measured chlorophyll concentrations collected on 8/3/22. Water sample BV analyses from this sampling date indicate dinoflagellate dominance, and the cross validation with higher resolution field spectra and Kilroy station spectra provides additional confidence that multi‐image Sentinel‐2 Components 4 and 5 are primarily associated with dinoflagellates. For further analysis, multi‐image Sentinel‐2 Component 4's dominant contributing factors are attributed to dinoflagellate and/or iron sulfate mineral with moderate confidence and Multi‐image Sentinel‐2 Component 5's dominant contributing factors are attributed to dinoflagellates and/or chlorite with moderate confidence. However, interference of suspended sediment in dinoflagellate identification is likely after high wind or rain events that increase turbidity.

Multi‐image Sentinel‐2 Component 6 identified as goethite was the least reliable match. It was necessary to reduce the probability of F to enter to 0.09 to find a match in the linear regression and the spectral match is *r* ^2^ = 0.711. This identification is supported by higher resolution IDs from the 10 nm field sampling and from the hyperspectral Kilroy data a sample measurement of the outside of an Ohio concretion that was identified as goethite based on reflectance and XRD measurements. This validation is indicative of goethite identification. Multi‐image Sentinel‐2 Components 4, 5, and 6 only account for 15.6% of image variance shown in Figure 3. It is likely that multi‐image Sentinel‐2 Component 6 is likely a suspended sediment associated with goethite. Further research is needed to fully understand the validity of suspended sediment minerology.

### 4.3 Interpretation of Component Identification and Field Sampling

Identification of brown, stressed *Thalassia* in both the satellite resolution and in the field ASD data is a significant discovery. When compared to a published map from the Saint Johns River Water Management District published in 2017 it shows remarkable similarities. “Brown, stressed *Thalassia*” in the spectral library is the spectrum for unhealthy seagrass digitized from Thorhaug et al. (2007). Seagrass is a vital resource in the IRL and an indicator of the overall health of the lagoon. Multi‐image Sentinel‐2 Component 3 represents seagrass and produces maps of seagrass in the area with great accuracy. It can also indicate the clarity of the lagoon because the bottom signal disappears from the satellite images when the lagoon is too turbid to see the bottom. Seagrass is a primary food source for the endangered manatee population and having fast temporal resolution on mapping is critically important and saves money for conservation groups that currently track it using field collected data, which can take a full season to collect and construct a basin‐wide map for the Banana River or other sub‐basins of the IRL. In contrast, remote sensing analysis can map the entire region on a given, cloud‐free, low turbidity day.

Extracted pixel values from each component score map and Chl‐a data correlate significantly, indicating suspended sediment, seagrass, cyanobacteria, and dinoflagellates presence at each sampling site. Multi‐image Sentinel‐2 Components 3, 4, and 5 correlate positively and multi‐image Sentinel‐2 Components 1, 2, and 6 correlate negatively. The same relationship occurs in TAL. This is likely due to multi‐image Sentinel‐2 Components 4, 5, and 6 being related to dinoflagellates and their dominance during this day and seagrass was likely visible at these locations due to the negative relationship with suspended sediment related components. More data is needed to quantify the relationship between bloom development and suspended sediment.

Cyanobacteria are identified as a primary component in the remote sensing analysis although it was only a minor presence in the water sample BV data. The reason for this disparity is likely to be due to water samples being collected in a 3‐day period in 2022 at a small number of stations while the most significant cyanobacterial bloom occurred earlier in 2020. The cyanobacteria were not in significant abundance during the 2022 field sampling based on comparison with the Kilroy sample station time series data as well as the water BV data. This is also consistent with the multi‐image Sentinel‐2 Component 1 spatial patterns on 3 June 2022. *A. lagunensis* was not identified in the VPCA and cell count showed that there was little presence of the brown tide algae during field sampling, although it had been identified in previous work using both VPCA and field observations (Judice et al., 2020), documenting that VPCA can effectively identify algae under temporally variable conditions.

It is interesting to note that there is elevated organic carbon wt.% at Grand South, and at OS2. Grand South sediment sample containing 4.5 wt.% TOC and OS2 2.9 wt.%. This is significant because a study conducted in Wang et al. (2022) showed positive correlations between the distribution of HAB species with TN, biogenic silica, and TOC, suggesting that organic matter originating from aquaculture activities and consequent high dinoflagellate productivity, shown in Figure 2 as Miozoa, may have a significant influence on the distribution of HAB species and facilitate the occurrence of HABs. More research is needed to fully understand the relationship between TOC, TN, and initiation of HABs within the IRL.

The water BV data represented in Figure 2 provide additional validation to the interpretation of the satellite remote sensing data. The water BV was dominated by dinoflagellate (Miozoa) in most areas except for Barge Junction, which was dominated by diatoms or cryptophytes, an observation consistent with the Optical Kilroy data from Barge Junction. This difference in the algal and cyanobacterial community at Barge Junction could be due to the heterogeneity in that area. Barge Junction is the canal connection between the northern IRL and the Banana River and so it is likely that it represents two different water bodies on the two sampling days during field work. The Barge Junction samples also had the smallest BV in the sampling data set which could influence the percentage representation of each algae division. The sediment sample at Barge Junction also had a very high C:N ratio indicating it is heavily influenced by terrestrial environments. These observations are consistent with its location in the canals. From a general seasonal perspective, diatoms frequently dominated blooms during colder months (i.e., November–March) in the Northern IRL and Banana River Lagoon, while picocyanobacteria and dinoflagellates typically dominated blooms during warmer months (i.e., April–October) (Phlips et al., 2015). The overall domination of dinoflagellates during the summer sampling event in August is consistent with this assessment.

### 4.4 Seagrass Population Assessment

Figure 7 shows remarkably similar results of the seagrass‐related multi‐image Sentinel‐2 Component 3 score map compared to field validated inventory assessments of seagrass. This exemplifies the ability of this analysis to quantify the aerial extent of seagrass within the IRL. This analysis is important because seagrass population is a critical resource and has been called a “coastal canary” due to its quick response to anthropogenic stressors and seagrass decline can indicate a loss of refuge for commercially and recreationally important species like sea turtles and manatees (Morris et al., 2022). During the analysis Sentinel‐2 MSI's higher spatial resolution likely allowed for the seagrass signal to be observed. A seagrass related component was identified in field collected hyperspectral data, Sentinel‐2 single image, and Sentinel‐2 multi‐image time series analysis. Previous studies using Sentinel‐3 OLCI like Judice et al., 2020 and our own single day image did not identify seagrass as a component and this is likely due to its significantly coarser spatial resolution. The reflectance signal among coarse spatial pixels was likely mixed too heavily with other water constituents to be derived as a component in a VPCA analysis. However, the ability to image seagrass extent is also dependent on turbidity, so when the water is more turbid the spatial patterns for multi‐image component 3 score maps are inconsistent or not present. This is because the water leaving reflectance signal does not reach the lagoon floor where seagrass primarily resides. Less seagrass has been shown to lead to increased turbidity and decreased light availability arising from resuspension of particles, which could make it more difficult for the seagrass to recover (Morris et al., 2021).

Through this study we have shown that this analysis accurately images the extent of seagrass on the lagoon floor. The utility of the multi‐image VPCA method is significant due to the ability for this process to be automated. The VPCA method can be continuously applied to new images and create new component score animations as fast as the satellites pass over. The ability to remotely estimate the extent of seagrass will cut the costs of monitoring aerial extent and allow field operations to be focused on causes and remediation. Dewberry was contracted by the SJRWMD to conduct the seagrass inventory used in Figure 7; it contained a total of 515 field points including 193 underwater video segments as well as aerial images taken over three days (Dewberry, 2018). The assessment involved aerial image acquisition, orthophotography, field work, and photo interpretation. The Sentinel 2 averaging VPCA code uses only open‐source technology (QGIS) and databases (European Space Agency, n.d.) yielding results in a very short amount of processing time. Interpretation of extent is still needed to create a shapefile assessment. Low turbidity days need to be selected to obtain an accurate representation of the extent of seagrass coverage. The VPCA assessment of seagrass is simple and fast in comparison to field or other aerial assessments.

### 4.5 Cyanobacterial Bloom Mapped by Phycocyanin Related Component

The multi‐image Sentinel‐2 Component 4 identified as dinoflagellates and the multi‐image Component 1 that we have identified as cyanobacteria show excellent agreement with a major *P. bahamense* dinoflagellate bloom observed in the summer, followed by a fall bloom of a nanoplanktonic cyanobacteria reported field biological cell count analysis in Phlips et al. (2021). In our component score maps we observed this relationship with the dinoflagellate component being prominent from January to September followed by replacement with the cyanobacterial component in the Fall represented in Figure 8. This shows the component analysis was able to identify and image a regime change between dinoflagellates and cyanobacteria in the IRL during our study. The cyanobacterial bloom identification is also consistent with the Kilroy field sensors' blue green algae measurements shown in panel 1 of Figure 8. The separation of blue‐green algae and Chl‐a count represents a dominance of cyanobacteria recorded at Kilroy field station Kars Park. Due to the agreement of published observation, field station data, and agreement in spatial pattern we believe component 1 accurately identified cyanobacterial bloom development.

The cyanobacterial bloom appears to initiate along the northwestern coastal waters of the banana river and within the northern IRL. Dinoflagellates over the 3‐year period showed a pattern of higher concentration in the coastal waters of the Banana River Lagoon. Cyanobacteria, and dinoflagellate blooms initiating in coastal waters and growing to the rest of the lagoon is an important observation and will need to be explored in future research to identify potential causes. The ability to map the extent, and development of different algae species will be critical for monitoring the ongoing health of the IRL as well as to identify potential causes of HABs in the future.

## 5 Conclusions

Although there were ambiguities among some of the component loading identifications for minerals due to the relatively coarse spectral resolution of Sentinel 2A/B MSI sensor in comparison to Sentinel‐3 OLCI, when the identification criteria are broadened from individual clays to total suspended clay minerals, there is excellent agreement across different spatial and spectral resolutions in the data sets explored. We note that given the low band count of the Sentinel 2 MSI sensor that more research will be needed to better differentiate the minerology of suspended sediments for this low band count sensor. These ambiguities, however, do not affect the study's main objective of identifying and mapping micri‐ and macro‐algae through time and improving spatial resolution. By conducting robust ground validation, we adequately address the limitations of Sentinel 2 A/B MSI sensor to yield a robust tool for mapping micro‐ and macro‐algae. Given the incredible differences in data volume (Sentinel‐2 average of 109 images, Sentinel‐3 single image VPCA, Sentinel‐2 single image VPCA, Field Hyperspectral data) the component identifications are remarkably similar. This was further supported by water BV data and the dominance of dinoflagellate during field sampling. The VPCA method's six retained components accounted for 96.7% of the total variance. This allowed for accurate interpretation of water quality characteristics by mapping extent of different components. The enhancement in the quality of identification using hyperspectral data in this study documents the importance of continued development and funding support for hyperspectral orbital sensors such the NASA EMIT, PACE and proposed NASA SBG missions.

We were able to differentiate cyanobacteria (component 1 *r* ^2^ = 0.865) from dinoflagellates (component 4 and 5 *r* ^2^ = 0.986 and *r* ^2^ = 0.978), and most notably identify and map seagrass extent with confidence (component 3 *r* ^2^ = 0.916). These observations were further supported by comparisons across sensors, optical Kilroy spectra, field collected data and previously published biological observations. This demonstrates that the VPCA method can identify optical components in an ecosystem that is evolving with time as well as compare VPCA across sensor resolutions. It was possible to determine micro and macro algae related components present in the component score maps accurately and with great confidence. Component maps were limited in accuracy during high turbidity events and represent a limitation of time series analysis and algae bloom identification. Spatial mapping and differentiation of algae species along with automated processing will enable local officials and non‐profits to benefit from easy to interpret component score maps. We hope that the supporting GitHub release in the Open Research section will serve as a roadmap for future water quality monitoring efforts. The analysis has been validated for this time period and location and we hope that it will be used for further monitoring of the IRL. The processing code can be adapted for other coordinates and analysis on other water bodies, but more research will be needed to understand its performance in conditions outside of a coastal lagoon system. By enabling fast mapping and identification of water quality components it alleviates the burden of extensive field sampling, allowing for more resources to be allocated toward identifying and addressing root causes of water quality decline.

## Conflict of Interest

The authors declare no conflicts of interest relevant to this study.

## Erratum

The supporting information in the originally published version of this article contained errors. The errors do not affect the main results or conclusions of the study. The supporting information has been corrected, and this may be considered the authoritative version of record.

## Supporting information

Supporting Information S1

## Data Availability

The Supporting Information S1 data set and code used for the VPCA analysis in the study are available at Zenodo via https://doi.org/10.5281/zenodo.22021520 with open access under Zenodo standard licensing (macwoodman, 2026).
