# Supplementary material for: Improving Micro– and Macro–Algae Speciation Mapping for Management: A Field‐Validated Sentinel‐2 and Sentinel‐3 Intercomparison in the Indian River Lagoon
Source: Geohealth. 2026 Oct 18;10(10):e2026GH001817. doi: 10.1029/2026GH001817 (PMC13677071; doi:10.1029/2026GH001817)
Supplement: Supplementary file 1 [file GH2-10-e2026GH001817-s001.docx]

*GeoHealth*

Supporting Information for

**Improving micro and macro–Algae Speciation Mapping for Management: A Field-Validated Sentinel-2 and Sentinel-3 Intercomparison in the Indian River Lagoon**

**McKenzie L. Woodman^1,2^, Joseph D. Ortiz^2^, Edith. A. Widder^3^ and Warren H. Falls^3^**

^1^Spectral Evolution Inc., Haverhill, MA

^2^Earth Sciences Department, Kent State University, Kent, OH

^3^Oceanic Research and Conservation Association, Vero Beach, FL

**Contents of this file**

Text S1 to S6

Figures S1 to S14

Table S1 to S1

**Introduction**

This document includes additional materials and analysis that support the methods, validation, and interpretation of the associated manuscript. These figures were generated to include supplementary information to maintain reproducibility and transparency. The graphs and figures included here are not directly necessary to understand the methods and conclusions of the analysis, but they document further analysis that was conducted to explore the resulting data as well as more detailed descriptions of methods.

Text S1.

This is a supplementary text for methods of field data and Kilroy data processing. Cell count samples were collected in 125ml HDPE plastic bottles. Samples were kept on ice in the field, then 10ml (5%) Lugol’s solution was added as a preservative and the samples were stored cold until received at BSA Environmental Services.

The field spectroradiometer was calibrated using a white Spectralon™ reference plate, attached to a ~2m photographic monopod equipped with a shoulder strap and used as a boom. The instrument was extended out over the side of the boat to collect reflectance and irradiance data away from the boat to avoid adjacency effects. Each individual spectrum collected by the instrument is an average of 30 spectral measurements. During the procedure at least three sets of 8 measurements yielding 24 or more reflectance measurements were collected at each site. The valid readings from each site are averaged together. Thus, each average reflectance spectra and its derivative at each site is based on up to 30x3x8 = 720 individual spectra. The same averaging procedure was used to collect irradiance and shaded irradiance using the RCR cosine theta receptor. Irradiance measurements were measured both shaded and unshaded so the diffuse to global ratio (a measure of cloud cover) can be calculated at each site.

Table S1. Shows the different spatial, temporal, and spectral resolutions of the spectral sensors used for this study.

Figure S1. Derivative Reflectance readings collected August 1st, 2022 with a handheld hyperspectral sensor in the at Barge Junction in the Indian River Lagoon, Florida interpolated to 10nm resolution, Sentinel-2 resolution, and Sentinel-3 resolution to represent the loss of information when using Sentinel-2 spectral readings. The green represents the 10nm resolution and the blue and orange represent the Sentinel-2 resolution and Sentinel-3. There are 24 spectral measurements represented in each average line here. Text S2.

The cell count data collected at the 6 sample sites is represented in Figure 2, 3, and 4. Figure 3 represents the ID of the division and the sum of BV um^3^/L. The total BV was greatest at Sykes Creek on 8/1/22 and at Grand South on the second sampling day. Sykes Creek, OS2, and Grand South have significantly different BV between sampling days.

Figure S2. sediment CaCO3, TIC, TC, TOC, TN and C: N at all field sampling locations.

Figure S3. Sediment TN at all field sampling locations.

Text S3.

Sediment samples were collected at all sampling sites on 8/3/22. The total organic carbon (TOC) has a range of 3.9 wt.% from 0.9 wt.% to 4.6 wt.%. The highest recorded TOC was at Grand South and the lowest recorded at Sykes Creek. Total inorganic carbon (TIC) has a range of 3.7 wt.% from 0.8 wt.% to 4.5 wt.%. The highest recorded TIC was at OS 2 and the lowest at Sykes Creek. They had relatively even C:N ratios, averaging X across the sampling sites except for Barge Junction which has TN under detectable limits indicating a very high C:N ratio. The highest recorded C:N ratio was at Barge Junction at Y and the lowest was at Grand South at Z. CaCO3 wt.% has a range of 30.6% from 6.6 wt.% to 37.2 wt.%. The highest wt.% recorded at OS2 and the lowest recorded at Sykes creek. The Total Carbon (TC) has a range of 6 wt.% from 1.4 wt.% to 7.4 wt.%. The highest recorded at Grand South and the lowest recorded at Sykes Creek. The Total Nitrogen (TN) has a range of undetectable (<w wt.%) to .49 wt. %. The highest recorded at Grand South and the lowest recorded at Barge Junction.

Figure S4. Sentinel-2 multi-image VPCA image dates.

Figure S5. Average Correlation calculated from the subset of images from the years 2020-2022. The images were filtered for 10 percent cloud coverage and for sun glint or sensor error. This yielded from processing 109 images.

Figure S6. Communality shows the proportion of variance shown by the components. Components 1-3 account for the majority of variance and 4-6 account for less. When added together they account for nearly all the image variance.

Figure S7. Component loadings showing the spectral shape of each component with the red edge region highlighted.

Text S4.

An average correlation matrix was calculated from a subset of images over a 3-year period 2020-2022 (109 images). The component graphs show the spectral shape of the average components and a red box overlayed for ease of identifying red edge responses, and an indication of a pigment-related component.

Figure S8. Graphs comparing Z scores of component loadings to their matched constituents. Column A represents z scores of average VPCA components, and their identification truncated to 5 bands. If they are a mineral column B extends the matched constituent to 9 bands.

Figure S9. Graphs showing Sentinel-3 VPCA Component Z scores from image collected on 8/03/2022

Figure S10. Graphs showing Field collected ASD data collected at all field sampling locations analyzed using the VPCA Components and represented as Z scores from field collection on 8/03/2022

Figure S11. Graphs showing Sentinel 2 single image VPCA Components represented as Z scores from an image on 8/03/2022

Figure S12. Graphs showing optical Kilroy field station VPCA Components represented as Z scores from an image on 8/03/2022

Figure S13. Graph showing similarities between clay mineral spectra when interpolated to Sentinel-2 resolution.

Text S5.

The component loading z-scores can be compared to the matched spectra z-scores from the spectral library. The left column is the components truncated at 5 bands to match pigment related products. If the best match is a mineral, they are represented in the right column as well by extending the graph with all nine Sentinel-2 bands used.

Figure S14. Shows correlation between sampled Chl-a ug/L against extract pixel values of Sentinel-2 Multi-image VPCA components at sample locations. Uncertainties for the field observations were estimated based on the standard error of the estimate across sites.

Text S6.

This supports the cell count domination of dinoflagellates. Turbidity was not correlated to Chl-a in these samples, and each component was not correlated to each other. Components 1 and 2 have the highest correlation but all components have a correlation R² > 0.9. This shows that all components are present at the sample sites. All components correlate with Chl-a, and TAL PE. TAL PE represents Total Algae-Phycoerythrin. None of the components correlate with the rest of the sonde metrics.

The spatial pattern in the component 1 map and the values of field measures of Chl-a are highly correlated. The spatial pattern shows that there was high concentration of phycocyanin in the coastal waters of IRL and around the bridges separating parts of the lagoon. Analysis of component 2 shows steady concentration throughout the lagoon, representing that phycocyanin was not everywhere that kaolinite was. Both are present to the same degree at each of the sampling points.
